# Response to Letters to the Editor Re: “Comparative Effectiveness of 2 Next-Generation Scatter Radiation Shielding Systems”

**DOI:** 10.1016/j.jscai.2025.104071

**Published:** 2025-12-11

**Authors:** Robert F. Riley, Stephen Kidd, Brian Stegman, Thom G. Dahle

**Affiliations:** Division of Cardiology, Overlake Medical Center, Bellevue, Washington; Division of Cardiology, St. Cloud Hospital, St. Cloud, Minnesota; Division of Cardiology, St. Cloud Hospital, St. Cloud, Minnesota; Division of Cardiology, St. Cloud Hospital, St. Cloud, Minnesota

We thank Dr Davies, Dr Cox, Dr Rizik, and Mr Smith for their thoughtful commentary on the importance of methodological rigor in comparative radiation protection studies. We share their commitment to high scientific standards, transparency, and the advancement of solutions that safeguard physicians, staff, and patients from occupational radiation exposure; however, several points raised in their letters require further clarification.

## Completeness of system evaluation

The letters assert that our study evaluated the EggNest Complete system (Egg Medical) (not the XR system, as mentioned in Dr Davies’ letter to editor) in its entirety, whereas the Rampart system (Rampart ic) was represented by only the Defender. This characterization is, unfortunately, misleading. The EggNest Complete is a fully integrated radiation protection platform—with table, overhead, and disposable components—that was commercially available and installed in hospitals at the time of the study (performed in August 2024). In contrast, the only Rampart product commercially available and in clinical use during the study period was the Defender, which had been previously professionally installed at the study site; therefore, this system was appropriately chosen for evaluation. Rampart’s subsequent launch of modular products such as Bunker, Shadow, Guardian, and Sentry do not diminish the validity of evaluating the Defender as the company’s primary commercial offering at the time of the study. Evaluating devices as they are most widely used in practice is not a limitation; rather, it is essential to producing clinically relevant findings regarding commercially available equipment.

## Methodological rigor and clinical relevance

The methods used in this clinical study followed the procedures outlined in a recent publication in *JSCAI*, which included the expertise of 3 radiation physicists.^1^ It is one of the most comprehensive protocols for evaluating enhanced radiation protection devices to date. Although the commentary criticizes the averaging of scatter fields, this approach is consistent with long-standing methods in radiation safety literature and provides practical, interpretable outcomes. Most importantly, identical methodology was applied across devices, ensuring fairness and internal validity.

The assertion that the Defender may have been misconfigured contradicts the study documentation. All devices were deployed per manufacturer instructions, and the experimental setup was transparently illustrated in the publication. Comparative research must evaluate systems not only under idealized conditions but also in ways that reflect real-world workflows.

## Acknowledgment of product evolution

We appreciate the recognition of EggNest’s recent advancements, including the Complete system and disposable shielding improvements; however, it is important to note that the Rampart Guardian and Sentry products referenced in the commentary were not commercially available at the time of study design and execution. Including unreleased or unvalidated devices in comparative research would undermine scientific rigor and risk conflating evidence with marketing claims.

## Transparency and independence

The study was conducted according to rigorous scientific standards, transparently reported, and peer-reviewed, with methods described in sufficient detail to permit replication. The presence of appropriately reported conflicts of interest does not diminish the study’s validity when proper safeguards, independent oversight, and peer review are in place. The same logic could be applied to several authors of the letters to the editor given that they have stated conflicts with Rampart.

## The question of premature comparisons

Dr Rizik argues that head-to-head comparisons between emerging devices are premature and that the standard of care—lead aprons and table shields—should remain the sole comparator. Although it is true that lead and table shields are still widely used, progress in occupational safety depends on direct comparisons between new technologies. Such studies help clinicians distinguish meaningful innovations from incremental ones and accelerate the adoption of solutions that deliver real improvements in protection.

## Conclusion

Radiation safety in the interventional suite is too important to be guided by speculation or selective product narratives. The study in question provided transparent, fair, and clinically relevant data on systems as they were commercially available and used in practice at the time of the study. As technologies evolve, future studies will expand these comparisons, but clinicians deserve clear, evidence-based insights into the solutions available to them today.

## Declaration of competing interest

Robert F. Riley serves as an advisor for Egg Medical, the sponsor of the study. The other authors reported no financial interests.
